# Magnetic Resonance Imaging Features on Deep Learning Algorithm for the Diagnosis of Nasopharyngeal Carcinoma

**DOI:** 10.1155/2022/3790269

**Published:** 2022-05-25

**Authors:** Ruijie Huang, Zhanmei Zhou, Xintao Wang, Xiaohua Cao

**Affiliations:** ^1^Department of Otorhinolaryngology, Jingdezhen Second People's Hospital, Jingdezhen 333000, Jiangxi, China; ^2^Department of Image, Jingdezhen Second People's Hospital, Jingdezhen 333000, Jiangxi, China; ^3^Department of Otorhinolaryngology, Ganzhou People's Hospital, Ganzhou 341000, Jiangxi, China

## Abstract

The objective of this research was to investigate the application values of magnetic resonance imaging (MRI) features of the deep learning-based image super-resolution reconstruction algorithm optimized convolutional neural network (OPCNN) algorithm in nasopharyngeal carcinoma (NPC) lesion diagnosis. A total of 54 patients with NPC were selected as research objects. Based on the traditional CNN structure, OPCNN was proposed. Besides, MRI processed by the traditional CNN model and the U-net network model was introduced to be analyzed and compared with its algorithm. The used assessment parameters included volume transfer constant (*K*^trans^), rate constant (*K*_ep_), volume fraction (*V*_e_), and apparent diffusion coefficient (ADC). The results showed that the values of Dice coefficient, peak signal-to-noise ratio (PSNR), and structural similarity (SSIM) of the OPCNN algorithm were significantly higher than those of the traditional CNN model and the U-net network model. Meanwhile, the difference was statistically significant (*P* < 0.05). *K*^trans^, *K*_ep_, and *V*_e_ in tumor lesions were significantly higher than those in the healthy side, while the ADC was significantly lower than that in the healthy side (*P* < 0.05). The sensitivity, specificity, and accuracy of dynamic contrast-enhancement magnetic resonance imaging (DCE-MRI) in the diagnosis of nasopharyngeal carcinoma staging were slightly higher than those in T2-weighted imaging (T2WI) and diffusion-weighted imaging (DWI). The diagnostic sensitivity of DCE-MRI was more than 85%, its diagnostic specificity was more than 75%, and its diagnostic accuracy was more than 90%. The AUC area of NPC diagnosed by combination of the three was significantly different from that diagnosed by single T2WI, DWI, and DCE-MRI (*P* < 0.05). The diagnostic accuracy of MRI based on the OPCNN algorithm for nasopharyngeal carcinoma (93.2%) was significantly higher than that of single MRI (76.4%). In summary, the OPCNN algorithm proposed in this study could improve the quality of MRI images, and the effect was better than the traditional deep learning model, which had the value of clinical promotion. The application value of DCE-MRI in the diagnosis of pathogenic lesions of nasopharyngeal carcinoma was better than conventional MRI. The combined application of T2WI, DWI, and DCE-MRI in the screening of nasopharyngeal carcinoma lesions could greatly improve the diagnostic accuracy of nasopharyngeal carcinoma.

## 1. Introduction

Nasopharyngeal carcinoma is a malignant tumor occurring on the top and sidewalls of the nasopharynx. It is one of the most frequent malignant tumors in China. The incidence rate is the first of the otorhinolaryngology malignant tumors. The lesions of nasopharyngeal carcinoma can be nodular, ulcerative, and submucosal invasive. The pathological type is mainly squamous cell carcinoma, and the other types are relatively rare [1–3]. Clinically, it is considered that the occurrence of nasopharyngeal carcinoma is mainly related to Epstein–Barr virus (EBV) infection, genetic factors, chemical factors, and environmental factors. In addition, individual factors also include long-term smoking, long-term drinking, and eating pickled food [4]. There are obvious gender and ethnic differences in nasopharyngeal carcinoma. Among them, the incidence rate of the yellow race is the highest, and the incidence rate of the black race is lower than that of the yellow race. The incidence rate of the white race is the lowest. Besides, the incidence rate of men is two times higher than that of women [5, 6]. Nasopharyngeal carcinoma grows in the nasopharynx behind the nasal cavity. Its location is hidden, and it is often asymptomatic in the early stage, which is easy to be ignored. With the development of the disease, patients may have symptoms such as tinnitus, deafness, hearing loss, nasal congestion, headache, facial paralysis, cranial nerve paralysis, and distant metastasis. Most patients were diagnosed only after finding neck mass or other metastatic symptoms, so they lost the best time for the treatment [7–9]. Therefore, to achieve early diagnosis and timely treatment, the early signal of nasopharyngeal carcinoma needs attention and vigilance.

There are many clinical diagnostic methods of NPC, such as physical examination, nasopharyngeal endoscopy, EBV serum examination, magnetic resonance imaging (MRI), computed tomography (CT), and positron emission tomography-computed tomography (PET-CT) [10]. Complete physical examination, especially examinations on 12 pairs of cranial nerves and lymph nodes of the neck, can effectively affect the therapeutic effects on NPC. Pharyngorhinoscopy can detect patients' lesser tubercles, granulomatous eminence, or frequent hemorrhage. EB virus is closely related to the incidence of NPC and can be adopted as the effective index of the adjuvant diagnosis of NPC [11, 12]. However, the above laboratory examination operations are complicated, and the accuracy of the results fluctuates greatly. In terms of imaging, chest CT is suitable for patients aged above 50, and they can effectively display lung metastasis or mediastinal lymph node metastasis among patients [13, 14]. PET-CT is suitable for patients with late NPC, and it can clearly show neck lymphadenectasis or distant metastasis. MRI imaging is a widely applied imaging technology at present and is characterized by a wide scanning range, numerous parameters, noninvasion, and high-resolution soft tissue [15].

Deep learning is a new research direction of machine learning. It is a series of algorithms created by data processed and decision-making mode created by the human brain [16, 17]. Convolutional neural network (CNN) is a feedforward neural network (FNN), which usually includes data input layer, operation layer, activation layer, pooling layer, and fully connected layer. It is a neural network. Convolution operation replaces traditional matrix multiplication operation. CNN can recognize the spatial relationship between data very well and show good application prospect in image classification, video recognition, and medical image processing [18]. The traditional CNN model adopts the convolution kernel method to extract features. Each convolution kernel represents a network training operator. Feature extraction is a digital process. The original image is transformed into the model for recognition and processing to obtain the most effective image features. However, its advantage is the attraction of extra parameters to increase the computation amount. The MRI image contains very rich and subtle structural information. The computation amount of algorithm is huge, and the operation lasts long when the traditional CNN model is used. Hence, a multipath dense connection structure (MDCS) was proposed to optimize the operation of feature extraction and the traditional CNN algorithm. Besides, the image super-resolution reconstruction algorithm optimized CNN (OPCNN) algorithm was designed and combined with the MRI image to investigate the diagnosis of NPC lesions, which is expected to provide help for clinical imaging diagnosis.

Deep learning is a new research direction of machine learning. It is a series of algorithms born by human brain processing data and creating patterns for decision-making. Convolutional neural network (CNN) is a feedforward neural network, which usually includes the data input layer, operation layer, activation layer, pooling layer, and fully connected layer. It is a neural network in which convolution operation replaces traditional matrix multiplication operation. CNN can well-identify the spatial relationship between data and has good application prospects in image classification, video recognition, medical image processing, etc. Therefore, this study combines the deep learning algorithm and MRI images to study the diagnosis of nasopharyngeal carcinoma lesions, in expectation to provide help for clinical imaging diagnosis.

## 2. Materials and Methods

### 2.1. Research Object

Fifty-four patients with nasopharyngeal carcinoma treated in the hospital from March 15, 2019, to July 20, 2021, were collected in this study, comprising 37 males and 17 females. All patients participated voluntarily and signed informed consent before the implementation of the project. All the contents in the research had been approved by ethics committee of the hospital.

Inclusion criteria were stated as follows: (1) patients with squamous cell carcinoma diagnosed by pathology; (2) patients who had not received radiotherapy and chemotherapy; (3) patients without metal dentures; (4) patients who voluntarily signed informed consent; (5) patients with good inspection compliance.

Exclusion criteria were stated as follows: (1) patients with contraindications to MRI; (2) patients with a history of drug allergy; (3) patients with surgical treatment; (4) patients with mental diseases; (5) patients with incomplete clinical data.

### 2.2. Magnetic Resonance Imaging Examination Method

3.0T superconducting magnetic resonance scanning equipment was used in the study. The patient was placed in the supine position on the examination table, the patient's nose tip was placed in the center of the head and neck coil, and the laser positioning line was located at the nose tip for routine MRI and dynamic MRI enhanced scanning.

Conventional MRI: (1) axial fat-suppressed T2-weighted imaging (T2WI) sequence: repetition time, 7500 ms; echo time, 90 ms; visual field, 230 × 230 mm; layer spacing, 1.2 mm; layer thickness, 5 mm; and excitation time, 3 times; (2) axial diffusion-weighted imaging (DWI): repetition time, 3200 ms; echo time, 80 ms; visual field, 230 × 230 mm; layer spacing, 1.2 mm; layer thickness, 5 mm; and excitation time, 10 times.

Dynamic MRI enhancement (DCE-MRI): the contrast agent was gadopentetic-diethylenetriamine pentaacetic acid (Gd-DTPA) (specification: 100 mL/bottle; flow rate: 3 mL/s; dose, 0.2 mmol/kg; and fire rate, 2.1 mL/s). Axial 3D-T1 fast gradient self-selected echo sequence: repetition time, 3.52 ms; echo time, 1.42 ms; visual field, 230 × 230 mm; layer spacing, 1.2 mm; layer thickness, 5 mm; and excitation time, 1 time.

Each sequence of images was sent to the workstation for processing. The quantitative parameters of permeability and tumor focus were measured as follows: *K*^trans^ (the amount of contrast medium entering the extracellular space from blood per unit volume of tissue in unit time), *K*_ep_ (the amount of contrast medium entering the blood vessel from the extracellular space in unit time), *V*_e_ (the volume of extracellular space in unit volume of tissue), and the apparent diffusion coefficient (ADC) value.

### 2.3. Image Super-Resolution Reconstruction Algorithm Based on Optimized Convolutional Neural Network

Conventional neural network (CNN) was generally composed of the input layer, convolution layer, and activation layer. The convolution operation in the convolution layer could be regarded as the inner product operation of image and convolution kernel. It is assumed that the sliding step size is *l* , the convolution kernel size is *r*, the boundary filling is *u* , and the input image size is *p∗q*. Equations (1) and (2) could be obtained as follows:

(1) $$\begin{array}{c} p^{\prime }=\frac{\left(p-r+2u\right)}{l}+1, \end{array}$$

(2) $$\begin{array}{c} q^{\prime }=\frac{\left(q-r+2u\right)}{l}+1. \end{array}$$

Here, *p*′ × *q*′ indicates the size of the output feature map. ReLU activation function was used by the activation layer to complete the mapping of features, introduce nonfeature expression for features, and enhance the expression ability of the network. The expression of ReLU activation function could be expressed as

(3) $$\begin{array}{c} h\left(x\right)=\left\{\begin{array}{cc} 0, & x<0,\\ x, & x\geq 0. \end{array}\right. \end{array}$$

**Figure 1:**
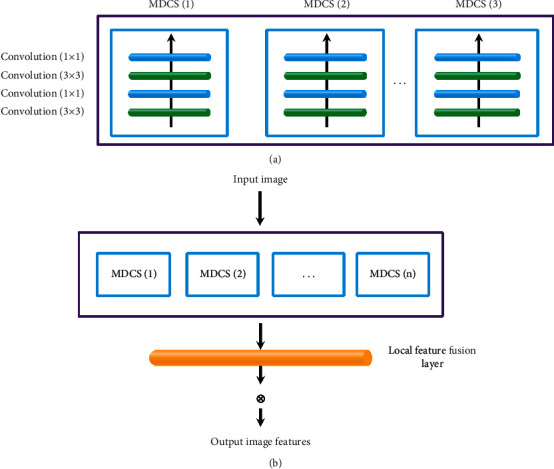
MDCS framework. (a) Schematic diagram of the multipath dense convolution block. (b) Schematic diagram of the multichannel dense connection structure.

The feature extraction part was a digital process that could be recognized and processed by converting the original image into a model to obtain the most effective image features. The traditional CNN model used the convolution kernel method for feature extraction, and each convolution kernel represented an operator for network training, to finally obtain richer image features, but the disadvantage was that it would attract additional parameters and increase the amount of calculation. It was considered that there was very rich and subtle structural information being contained in MRI images. Thus, a multipath dense connection structure (MDCS) (Figure 1) was designed in this study to optimize the feature extraction operation.  Figure 1(a) shows a multipath dense convolution block. It could be observed that the size of the convolution block was 2 × 2 and 4 × 4.  Figure 1(b) reveals the complete MDCS framework.

For the multipath dense convolution block, the image features extracted at layer *j* could be expressed as

(4) $$\begin{array}{c} H_{j}^{\prime }=\lambda \left[v_{j}\times \langle H_{0},H_{1},H_{2},\cdots H_{j-1}\rangle +c_{j}\right]. \end{array}$$

In (4), *H*_*j*_′ represents the image features extracted by layer *j*, 〈*H*_0_, *H*_1_, *H*_2_, ⋯*H*_*j*−1_〉 represents the sum of the output features of the input layer and the previous layer *j* − 1, *v*_*j*_ represents the weight of layer *j*, *c*_*j*_ represents the offset of layer *j*, and *λ*[] represents the activation function.

In addition, the adjustment of parameters was realized through a reverse neural network, which could lead to the problem of gradient disappearance. Therefore, this study also added local residual connection in multipath dense convolution blocks, which can be expressed as

(5) $$\begin{array}{c} H=H_{j-1}+H^{\prime }, \end{array}$$

which indicated that *H*_*j*−1_ and *H*′ could be quickly connected to reduce the computational complexity.

A multiway dense convolution block could greatly enrich the extracted image features, but it could also increase the parameters and produce many redundant features. Therefore, this study also introduced a local feature fusion block (LFFB) to reduce the dimension of image features. Then, the final output result could be expressed as

(6) $$\begin{array}{c} H^{\prime }=\lambda \left[v_{LFFB}\times \langle h_{0},h_{1},h_{2},\cdots h_{n-1},h_{n}\rangle +c_{LFFB}\right]. \end{array}$$

In (6), *v*_*LFFB*_ represents the weight of the local feature fusion block and *c*_*LFFB*_ represents the offset of the local feature fusion block. In this study, the image super-resolution reconstruction algorithm based on optimized CNN was set as OPCNN.

### 2.4. Image Evaluation Index

To analyze the performance of the OPCNN algorithm proposed in this study, the traditional CNN model and the U-net network model [19] were introduced as a comparison.

Dice coefficient, peak signal-to-noise ratio (PSNR), and structural similarity (SSIM) were used as indexes to evaluate the accuracy of segmentation results.

(7) $$\begin{array}{c} \text{Dice}=\frac{2TP}{\left(2TP+FP+FN\right)},\\ \text{PSNR}=10{\text{log}}_{10}\left[\frac{\left(\text{maximum pixel value}\right)2}{\text{MSE}}\right],\\ \text{MSE}=\frac{\sum_{i=1}^{M}\sum_{j=1}^{N}{\left(G{\;}^{*}-G\right)}^{2}}{M\times N},\\ \text{SSIM}=A_{1}\left(x,y\right)\times A_{2}\left(x,y\right)\times A_{3}\left(x,y\right),\\ A_{1}\left(x,y\right)=\frac{\left(2\eta_{x}\eta_{y}+a_{1}\right)}{\left(\eta_{x}{\;}^{2}+\eta_{y}{\;}^{2}+a_{1}\right)},\\ A_{2}\left(x,y\right)=\frac{\left(2\kappa_{x}\kappa_{y}+a_{2}\right)}{\left(\kappa_{x}{\;}^{2}+\kappa_{y}{\;}^{2}+a_{2}\right)},\\ A_{3}\left(x,y\right)=\frac{\left(2\kappa_{x}{\;}_{y}+a_{3}\right)}{\left(\kappa_{x}\kappa_{y}+a_{3}\right)}. \end{array}$$

TP represents true positive, FP represents false positive, FN represents false negative, *MSE* represents the mean square error, *G* ^*∗*^ and *G* represent two images, *M* × *N* represents image size, *η* represents image pixel value mean, *κ* represents image pixel standard deviation, *A*_1_(*x*, *y*) represents brightness similarity, *A*_2_(*x*, *y*) represents contrast similarity, *A*_3_(*x*, *y*) represents structural similarity, and *a*_1_, *a*_2_, and *a*_3_ were all parameters.

### 2.5. Statistical Methods

An SPSS19.0 version statistical software was used for data processing and analysis in this study. The measurement data were expressed by mean ± standard deviation, and counting data were expressed by percentage (%). One-way ANOVA was used for pairwise comparison. The difference was statistically significant (*P* < 0.05).

## 3. Results

### 3.1. Quality Comparison of Tumor Segmentation Results with Different Algorithms

**Figure 2:**
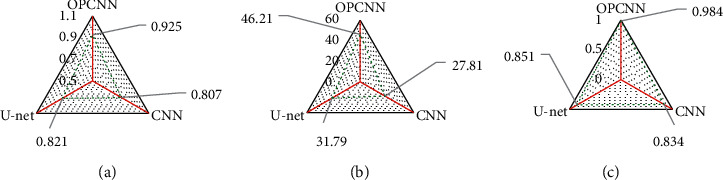
Quality comparison of tumor segmentation results of different algorithms. (a) Dice coefficient; (b) PSNR; (c) SSIM.  ^*∗*^ indicated that the difference was statistically significant compared with the OPCNN algorithm (*P* < 0.05).

Figure 2 shows the comparison of quality of tumor segmentation results of different algorithms. According to the results, Dice coefficients of the traditional CNN model, U-net network model, and OPCNN algorithm were 0.807, 0.821, and 0.925, respectively. PSNR of the traditional CNN model, U-net network model, and OPCNN algorithm reached 27.81, 31.79, and 46.21, respectively. SSIM of the traditional CNN model, U-net network model, and OPCNN algorithm amounted to 0.834, 0.851, and 0.984, respectively. The comparison showed that Dice coefficient, PSNR, and SSIM of the OPCNN algorithm were remarkably higher than those of the traditional CNN model and U-net network model. The differences demonstrated statistical significance (*P* < 0.05).

### 3.2. Image Features of Patients with Routine Magnetic Resonance Imaging

**Figure 3:**
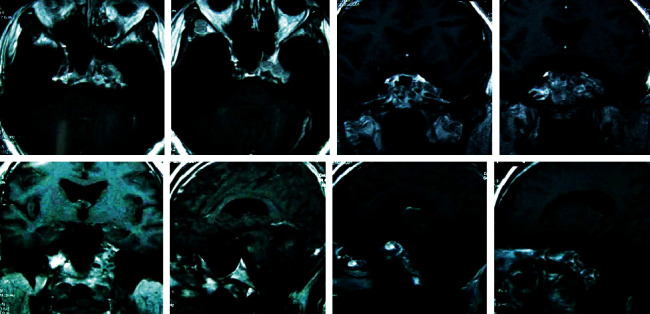
Conventional MRI scanning image of a patient with nasopharyngeal carcinoma.

Figure 3 gives the conventional MRI scanning image of a patient with nasopharyngeal carcinoma. It was suggested that there were irregular abnormal signals on the outer wall of the posterior wall of the top of the nasopharynx. The image shows that the eustachian tube and parapharyngeal space narrowed, the mass involved the left sphenoid sinus upward, the left nasal muscle space, and carotid sheath forward. There were soft tissue shadows.

**Figure 4:**
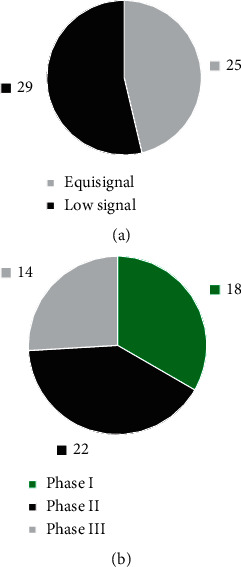
Routine MRI scanning diagnosis results of nasopharyngeal carcinoma. (a) MRI features. (b) Staging diagnosis results.

Figure 4 indicates that, among the 54 patients, 25 showed equal signal on T2WI image and 29 showed low signal; 18 cases of stage I, 22 cases of stage II, and 14 cases of stage III were diagnosed by routine MRI.

### 3.3. Results of Quantitative Parameters of Tumor Focus and Healthy Side of Patients

**Figure 5:**
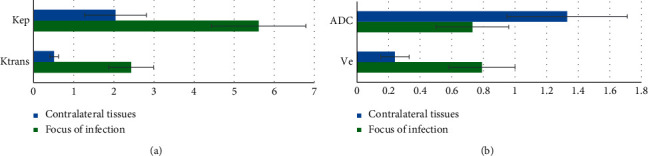
Comparison of quantitative parameters of tumor focus and healthy side of patients. (a) *K*^trans^ and *K*_ep_; (b) *V*_e_ and ADC.  ^*∗*^ indicated that the difference was statistically significant compared with the tumor focus (*P* < 0.05).

Figure 5 shows the comparison of quantitative parameters (*K*^trans^, *K*_ep_, *V*_e_, and ADC) between the tumor focus and the healthy side. It can be observed that *K*^trans^, *K*_ep_, and *V*_e_ in the tumor focus were significantly higher than those in the healthy side, and the difference was statistically significant (*P* < 0.05); ADC in the tumor focus was significantly lower than that in the healthy side, and the difference was statistically significant (*P* < 0.05).

### 3.4. Comparison of Diagnostic Effects between Conventional Magnetic Resonance Imaging and Dynamic Contrast-Enhanced Magnetic Resonance Imaging

**Figure 6:**
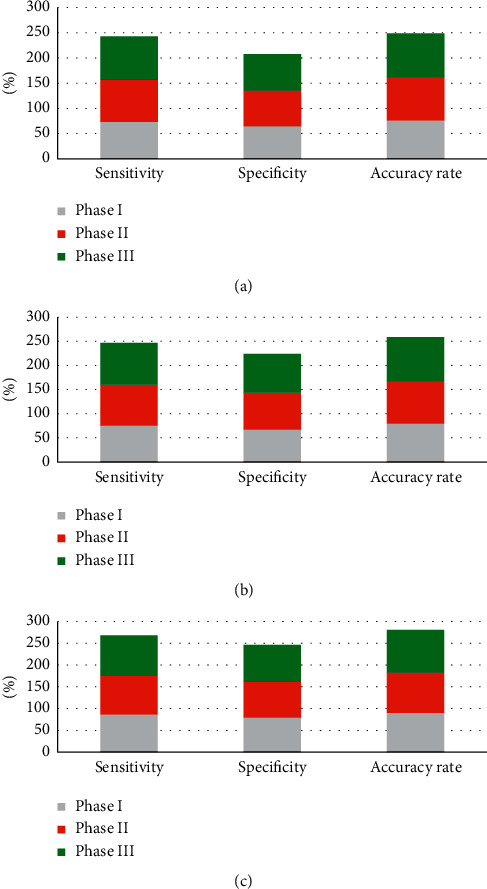
Sensitivity, specificity, and accuracy of T2WI, DWI, and DCE-MRI in the diagnosis of nasopharyngeal carcinoma stage. (a) The diagnosis result of T2WI for nasopharyngeal carcinoma stage; (b) the diagnosis result of DWI for nasopharyngeal carcinoma stage; (c) the diagnosis result of DCE-MRI for nasopharyngeal carcinoma stage; (d) the diagnosis result of T2WI, DWI, and DCE-MRI for nasopharyngeal carcinoma stage.

Figure 6 displays the comparison of the sensitivity, specificity, and accuracy of T2WI, DWI, and DCE-MRI in staging diagnosis of NPC. According to Figure 6, DCE-MRI showed high diagnostic sensitivity, specificity, and accuracy of NPC staging, reaching over 85%, 75%, and 90%, respectively.

### 3.5. Comparison of T2-Weighted Imaging, Diffusion-Weighted Imaging, Dynamic Contrast-Enhancement Magnetic Resonance Imaging, and Their Combined Detection in the Diagnosis of Nasopharyngeal Carcinoma

**Figure 7:**
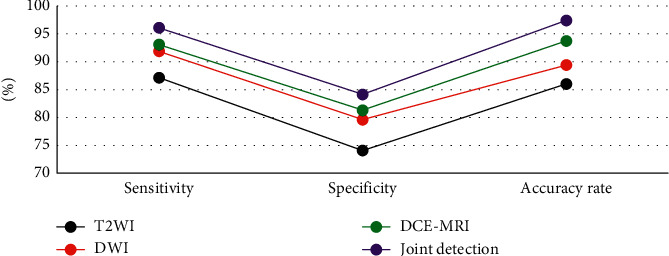
Comparison of T2WI, DWI, DCE-MRI, and their combined detection in the diagnosis of nasopharyngeal carcinoma.

Figure 7 shows the comparison of diagnostic effects of T2WI, DWI, DCE-MRI, and their combined detection on nasopharyngeal carcinoma. It was suggested that the diagnostic sensitivity, specificity, and accuracy of T2WI for nasopharyngeal carcinoma were 87.14%, 74.08%, and 86.03%, respectively. The sensitivity, specificity, and accuracy of DWI in the diagnosis of nasopharyngeal carcinoma were 91.86%, 79.61%, and 89.41%, respectively. The sensitivity, specificity, and accuracy of DCE-MRI in the diagnosis of nasopharyngeal carcinoma were 93.07%, 81.31%, and 93.73%, respectively. The sensitivity, specificity, and accuracy were 96.08%, 84.14%, and 97.41%, respectively. The sensitivity, specificity, and accuracy of the combined detection of the three were slightly higher than those of T2WI, DWI, and DCE-MRI, which were 96.08%, 87.14%, and 97.41%, respectively.

### 3.6. Receiver Operating Characteristic Curve Analysis

**Figure 8:**
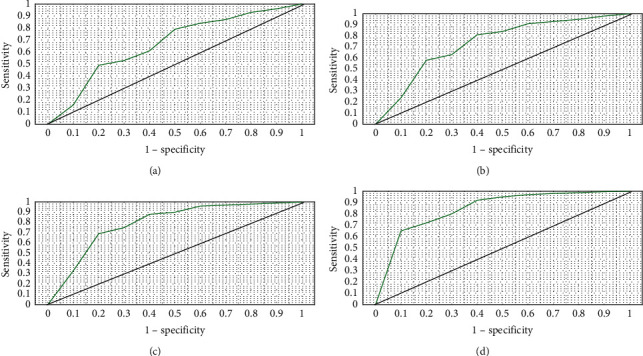
ROC curves of T2WI, DWI, DCE-MRI, and their combined detection. (a) T2WI; (b) DWI; (c) DCE-MRI; (d) their combined detection.

Figure 8 indicates that the AUC area of T2WI for nasopharyngeal carcinoma diagnosis was 0.833, DWI for nasopharyngeal carcinoma diagnosis was 0.851, DCE-MRI for nasopharyngeal carcinoma diagnosis was 0.891, and the AUC area of combined three for nasopharyngeal carcinoma diagnosis was 0.942. The analysis of variance suggested that the AUC area of NPC diagnosed by combination of the three was significantly different from that diagnosed by single T2WI, DWI, and DCE-MRI (*P* < 0.05).

### 3.7. Comparison of Diagnostic Performance between Magnetic Resonance Imaging Based on Optimized Convolutional Neural Network Algorithm and Single Magnetic Resonance Imaging

**Figure 9:**
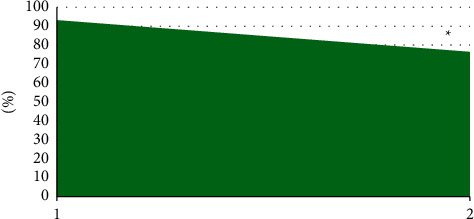
Comparison of diagnostic accuracy between MRI based on the OPCNN algorithm and single MRI.  ^*∗*^ indicated that the difference was statistically significant compared with MRI based on the OPCNN algorithm (*P* < 0.05).

Figure 9 illustrates that the diagnostic accuracy of MRI based on the OPCNN algorithm for nasopharyngeal carcinoma (93.2%) was significantly higher than that of single MRI (76.4%). The difference was statistically significant (*P* < 0.05).

## 4. Discussion

NPC is one of the conventional head and neck malignant tumors. Because its early symptom is not specific, the early diagnostic effect is usually unsatisfactory. Early detection of NPC lesions is beneficial to the clinical treatment effect and the prognosis of patients' quality of life [20–22]. At present, deep learning combined with the MRI technology is applied in tumor screening more significantly. The images obtained by different sequences show their respective advantages. Hence, a traditional CNN structure-based image super-resolution reconstruction algorithm OPCNN was proposed at first, and the traditional CNN model and U-net network model were introduced, compared, and analyzed.

The results revealed that Dice coefficient, PSNR, and SSIM of the OPCNN algorithm were all, obviously, higher than those of the traditional CNN model and U-net network model, and the differences showed a statistical meaning (*P* < 0.05). The results were consistent with that obtained by Ai et al. [23]. It was indicated that the segmentation effect of the OPCNN algorithm on MRI images was superior to that of the traditional CNN model and U-net network model, and it demonstrated application and promotion values.

Based on the analysis of the imaging features of conventional MRI and OPCNN algorithm-based dynamic enhanced MRI of 54 NPC patients, 25 cases showed equal signal on T2WI and 29 showed low signal on T2WI. The comparison of quantitative parameters of tumor focus and health side tissues suggested that *K*^trans^, *K*_ep_, and *V*_e_ of tumor focus tissues were significantly higher than those of healthy side tissues, while ADC was apparently lower than that of healthy side tissues. The differences were statistically significant (*P* < 0.05), which indicated that MRI quantitative parameters, including *K*^trans^, *K*_ep_, *V*_e_, and ADC could be viewed as the effective indexes of diagnosing NPC [24, 25]. In terms of single-sequence MRI images, it was found out in the research that the diagnostic sensitivity (over 85%), specificity (over 75%), and accuracy (over 90%) of DCE-MRI in the NPC staging were all slightly higher than those of T2WI and DWI. The results showed that the application effect of DCE-MRI in NPC diagnosis was superior to that of conventional MRI. The combination of multiple detection methods was also common in clinical disease diagnosis [26].

Besides, it was also shown in the research that the sensitivity, specificity, and accuracy of the three combined methods in NPC detection were all slightly higher than those of T2WI, DWI, and DCE-MRI alone (96.08%, 87.14%, and 97.41%, respectively). Analysis of receiver operating characteristic (ROC) curves revealed that areas under curves (AUC) of three combined diagnosis showed statistical meaning (*P* < 0.05) compared with those of T2WI, DWI, and DCE-MRI alone. Hence, the combination of T2WI, DWI, and DCE-MRI could enhance the accuracy of NPC diagnosis dramatically.

## 5. Conclusion

A traditional CNN structure-based image super-resolution reconstruction algorithm OPCNN was proposed and applied in the clinical dynamic enhanced MRI scanning on 54 patients with NPC. The result showed that the diagnostic accuracy of OPCNN algorithm-based DCE-MRI remarkably improved. However, the diagnosis of NPC of different pathological types was not further divided and analyzed due to the small sample size of the included patients. In addition, only T2WI, DWI, and DCE-MRI were analyzed in combination without investigating the paired combination of three sequences. In subsequent studies, it is necessary to include and deeply discuss more NPC case data from different sources. To sum up, the research results provided the references for the development of the clinical diagnosis of NPC.

## Data Availability

The data used to support the findings of this study are available from the corresponding author upon request.
